# Variations in adverse pregnancy and birth outcomes among Latin American and Caribbean-Born birthing people by region of origin, California birth cohort, 2007–2020

**DOI:** 10.1186/s12884-025-07483-6

**Published:** 2025-04-02

**Authors:** Salma Iraqi, Shira Goldenberg, Rebecca J. Baer, Hector Lemus, Gretchen Bandoli

**Affiliations:** 1 Department of Epidemiology and Biostatistics, School of Public Health, San Diego State University, San Diego, CA United States of America; 2 Department of Pediatrics and Herbert Wertheim School of Public Health, University of California San Diego, San Diego, CA United States of America

**Keywords:** Pregnancy and birth outcomes, Latin America and the Caribbean, U.S.-born, Disaggregation, Region of origin, Social determinants of health, Mediators

## Abstract

**Background:**

Although many studies have highlighted better pregnancy and birth outcomes among foreign-born Latinas than among U.S.-born people, few have assessed heterogeneity in outcomes disaggregated by region of origin. We examined adverse pregnancy and birth outcomes among birthing people born in Latin America and the Caribbean (LAC) compared to people born in the U.S.

**Methods:**

We used a retrospective cohort from the Study of Outcomes in Mothers and Infants compiled from California births (2007–2020). We examined descriptive statistics, unadjusted, and adjusted odds ratios for the association between LAC nativity and region of origin (versus U.S.-born) and preeclampsia, gestational diabetes, preterm birth, and small for gestational age. We also assessed the potential mediating roles of education, health insurance, and prenatal care.

**Results:**

The sample included 5,917,974 infants, with 3,555,173 born to U.S.-born birthing people, and 1,385,679 born LAC-born birthing people, with the vast majority being from Mexico (82%) and Central America (14%). The odds of each outcome among those from LAC regions were lower relative to U.S.-born individuals, with the following exceptions. The adjusted odds of gestational diabetes was higher among those born in Mexico (13.3% vs. 8.0%, AOR: 1.6, 95% Cl: 1.6–1.6) and Central America (11.1% vs. 8.0%, AOR: 1.3, 95% Cl: 1.3–1.3) compared to those born in the U.S. The adjusted odds of preterm birth was higher for those born in the Caribbean (8.5% vs. 7.2%, AOR: 1.1, 95% CI: 1.0-1.2) and Central America (8.0% vs. 7.2%, AOR: 1.1, 95% CI: 1.1–1.1) compared to the U.S. Similarly, the adjusted odds of small for gestational age were higher for those born in the Caribbean (10.5% vs. 9.2%, AOR: 1.2, 95% CI: 1.2–1.3) and Central America (10.4% vs. 9.2%, AOR: 1.2, 95% CI: 1.2–1.2). Education and health insurance were identified as mediators of the associations.

**Conclusion:**

There is significant heterogeneity in adverse pregnancy and birth outcomes among those born in LAC by region of origin, specifically among people from Mexico, Central America, and the Caribbean. These findings highlight the importance of assessing disaggregated data to address the distinct pregnancy and birthing needs of diverse foreign-born birthing people.

**Supplementary Information:**

The online version contains supplementary material available at 10.1186/s12884-025-07483-6.

## Background

Epidemiological studies examining pregnancy and birth outcomes of birthing people of Latin American origin in the U.S. have observed substantial variability in findings, with the majority of work suggesting potentially protective effects [1–4]. “Latin” typically refers to people from or descendent of Latin American and the Caribbean (LAC), a large and highly diverse region, including more than 650 million people, 33 countries, and a variety of racial, ethnic, linguistic, and socioeconomic groups [5–7]. Most immigrants in the U.S. and nearly half of California’s immigrant population originate from LAC [6]. While some studies have focused on pregnancy and birth outcomes among birthing people from specific LAC countries (e.g., Mexico, Puerto Rico), most collapse groups based on ethnicity or foreign-born status compared to U.S.-born birthing people, which may overlook significant heterogeneity in risk and needs among birthing people from LAC [1, 4, 8–16]. Life course theory posits that early life exposures, including historical, environmental, cultural, and systemic factors experienced in childhood, contribute to health and wellbeing in adulthood, including reproductive health. Structural factors that may differ by maternal birth country or region include socioeconomic factors, diet and nutrition, childhood adversity, stress, discrimination or inequality, environmental pollutants, presence or lack of coping resources or social support, and immigration experiences [17–27]. As these factors all influence reproductive health, aggregating across different countries or regions may mask this baseline heterogeneity.

Indeed, research has shown mixed results in pregnancy and birth outcomes among LAC-born birthing people, depending on their country or region of origin. For instance, a previous U.S. study observed that Mexican-born birthing people had a lower prevalence of preterm birth compared to U.S.-born non-Hispanic Whites [1]. However, those born in Puerto-Rico and El Salvador had a higher prevalence of preterm birth compared to Mexican-born birthing people [1]. Another study found that the prevalence of infants born small for gestational age (SGA) ranged from 3.9% for infants born to people from Bolivia to 9.7% for those born to people from Puerto Rico [28]. These findings suggest that there may be substantial heterogeneity in experiences based on region or subregion within LAC and indicate the need for population-based research to disaggregate analyses of pregnancy and birth outcomes where possible to generate more nuanced understanding and inform interventions addressing potential variation in outcomes and risks by subgroup. Additionally, few studies have looked at potential mediating effects, especially with regards to education, health insurance, and access to prenatal care, which have been shown to be associated with adverse pregnancy and birth outcomes in past studies and are likely to vary based on immigration experience [12, 29–40].

Given these gaps in the literature, we aimed to [1] describe variations in adverse pregnancy and birth outcomes (preeclampsia, gestational diabetes, preterm birth, and small for gestational age) by LAC nativity and by region of origin within LAC, and [2] assess the roles of education, health insurance, and prenatal care as potential mediators of these relationships.

## Methods

### Study design and population

We used data from the Study of Outcomes in Mothers and Infants (SOMI), an administrative birth cohort compiled from California birth records between 2007 and 2020. Birth certificates were probabilistically linked to hospital, emergency department, and ambulatory surgery records from the Department of Health Care Access and Information (HCAI) for both the mother and infant (one year before birth until the year after birth) [41]. The linked records provided diagnostic and procedure codes based on the International Classification of Diseases, 9th Revision (ICD-9), and 10th Revision, Clinical Modification (ICD-10) reported to HCAI by the hospitals. SOMI was approved by the University of California, San Diego (UCSD) and the Committee for the Protection of Human Subjects within the Health and Human Services Agency of the State of California.

**Fig. 1:**
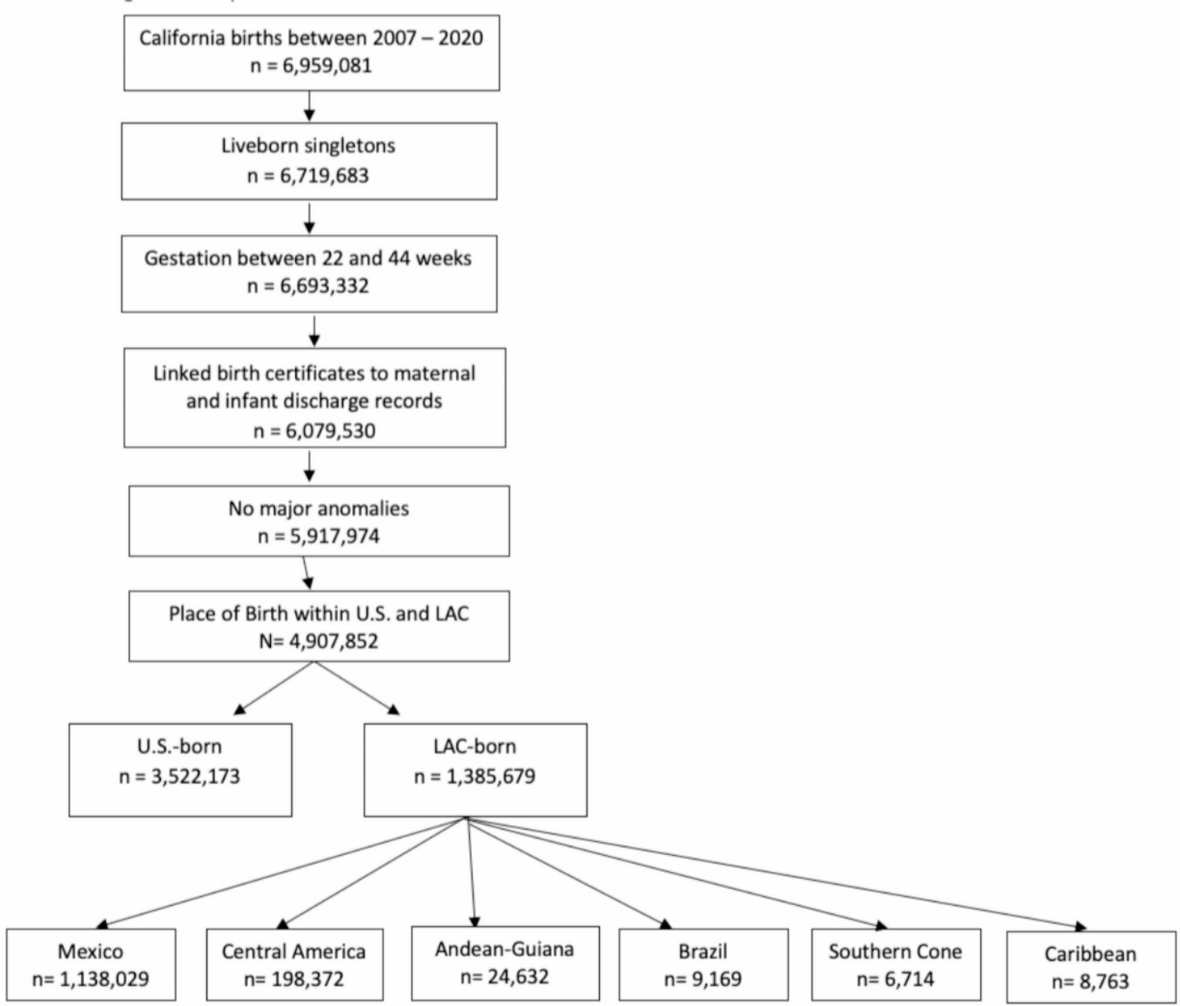
Study Population

From 2007 to 2020, there were 6,959,081 live births in California. For this analysis, we included singleton births between 22 and 44 weeks of gestational age, with successful linkage between vital statistics, and HCAI hospital discharge records. We excluded those who had births with anomalies (e.g., major structural birth defects, chromosomal abnormalities) or fetal deaths. We included only those born in the U.S. and LAC, excluding people born in other regions. The resulting analytic sample included 3,522,173 infants born to people from the U.S. and 1,385,679 infants born to people from the LAC (Fig. 1).

### Maternal characteristics

Variables to describe the sample were selected based on a literature review and data availability [33, 37, 41–43]. From birth certificates, we examined self-reported maternal race/ethnicity (non-Hispanic White, non-Hispanic Black, non-Hispanic Asian, Hispanic, non-Hispanic other), maternal age at delivery (years), infant year of birth (between 2007 and 2020), maternal education (less than high school, high school degree or GED, college or more, and missing), body mass index (BMI, kg/m^2^, calculated from pre-pregnancy weight and height), expected payer for delivery (private insurance, public insurance, other payment, missing), smoking in pregnancy and nulliparity (yes or no). We abstracted health behaviors during pregnancy reported from ICD codes in HCAI data: smoking, substance use disorder, and alcohol use disorder. Additionally, we assessed four levels for adequacy of prenatal careig (adequate plus, adequate, intermediate, and inadequate) using the Adequacy of Prenatal Care Utilization (APNCU) index [44]. Inadequate care starts after the 4th month or involves less than 50% of expected visits. Intermediate care starts by the 4th month with 50–79% of expected visits. Adequate care starts by the 4th month with 80–109% of visits, and Adequate-plus care starts by the 4th month with 110% or more of expected visits [44]. Finally, we included prepregnancy conditions ascertained from maternal HCAI records as a dichotomous variable for the presence or absence of diabetes and chronic hypertension. The data sources for outcomes, variables, and relevant ICD codes are provided in the supplemental Table 1.

### Measurement of exposures

Birthing people were classified into two groups based on their self-reported place of birth: U.S. (U.S.-born) and Latin America and the Caribbean (LAC-born). LAC-born people were disaggregated by region of origin, including Mexico, Central America (Guatemala, Honduras, El Salvador, Panama, Nicaragua, Belize, and Costa Rica), the Andean-Guiana Shield Regions (Venezuela, Columbia, Ecuador, Peru, Bolivia, Guyana, Suriname, and French Guiana), Brazil, Southern Cone (Chile, Argentina, Paraguay, and Uruguay), and the Caribbean (Anguilla, Antigua and Barbuda, Aruba, Barbados, Bahamas, Cayman Islands, Cuba, Dominica, Dominican Republic, Grenada, Haiti, Jamaica, Montserrat, Martinique, Puerto Rico, Caicos and Turks Islands, Tobago and Trinidad, British Virgin Islands, Virgin Islands, Saint Kitts-Nevis, Saint Lucia, Grenadines, and Saint Vincent). While acknowledging the distinctiveness of Guyana, Suriname, and French Guiana compared to other countries in Latin America, we opted to include them with the Andean-Guiana Shield region due to small sample and geographical proximity for this analysis. The U.S.-born comparison group included all birthing people across the 50 states, regardless of race or ethnicity.

### Measurement of outcomes

Maternal and infant diagnoses from health records were determined from any hospitalization or emergency department encounter during pregnancy and up to one year after delivery. Preeclampsia and GDM were identified through ICD-9 and ICD-10 codes from pregnancy, whereas preterm birth (defined as < 37 weeks) and SGA (defined as < 10th percentile; calculated from infant sex, gestational age, and birthweight per Talge 2014) were from birth certificate records [45].

### Statistical analyses

To characterize the sample, we summarized the frequencies of maternal characteristics and outcomes of interest by region of origin (i.e., LAC vs. U.S., disaggregated regions within LAC vs. U.S.). We used logistic regression models to derive odds ratios for the birthing peoples’ region of origin with 95% confidence intervals (95% CIs) for preeclampsia, GDM, preterm birth, and SGA. We fitted two models for each outcome: unadjusted odds ratios comparing adverse pregnancy and birth outcomes between LAC-born and US-born people, and models adjusted for maternal age and infant year of birth. For the second part of our analysis, we focused on LAC-born people, examining outcomes by different regions. Here we compared birthing people region of origin (LAC-born) with the U.S.-born birthing people, again in unadjusted models, and then in models adjusting for maternal age and infant year of birth. Separate models were completed for each outcome. Although age is not a strict confounder, we adjusted for it due to its association with birth outcomes and regional imbalances. Lastly, given the span of 2007–2020, the political context surrounding Latin American and the Caribbean immigration could have influenced both migration patterns and health outcomes. Adjusting for infant year of birth could help control for temporal variations and potential confounding effects.

### Mediation analyses

Sociodemographic characteristics and health related factors were not included as covariates in models, and they do not meet the definition of confounders (i.e. not a cause of nativity). Instead, these variables were subsumed into total effects estimates, and variables of interest were separately analyzed as mediators in the causal mediation analysis. For associations with odds ratios greater than 1.0, we explored candidate mediators that could explain the relationship between region of origin and pregnancy and birth outcomes, similar to previously published work [46]. Causal mediation analysis was performed using Valeri and VanderWeele’s SAS macro (‘%mediation’) [47]. Models were assessed for exposure-mediator interactions, and these interactions were included in models when significant. Candidate mediators were selected based on hypothesized sociodemographic or health access measures that may differ by country or region of origin (health insurance, prenatal care, and education) [12, 48–50]. Each mediator was modeled separately, with one mediator examined for each outcome at a time. For example, when modeling birthing people born in Mexico as the exposure, education as the mediator, and gestational diabetes as the outcome, we calculated the proportion mediated to quantify how much of the association between Mexico as the exposure and gestational diabetes as the outcome was explained by education.

## Results

### Descriptive results

#### U.S.-born compared to LAC-born

Our analysis included 5,917,974 infants, with 3,555,173 births to U.S.-born birthing people and 1,385,679 births to LAC-born birthing people. Among U.S.-born birthing people, 43.6% identified as Hispanic and 37.8% as non-Hispanic White, while 97.8% of LAC-born birthing people identified as Hispanic and less than 1% as non-Hispanic White (Table 1). The average maternal age was 28.5 years for U.S.-born and 29.3 years for LAC-born birthing people. BMI was similar between U.S.-born (26.7 kg/m2) and LAC-born (26.5 kg/m2), both falling in the overweight range (25.0–29.9 kg/m2). A higher proportion of U.S.-born birthing people smoked (3.74%), had alcohol use disorders (0.41%), and had other substance use disorders (2.66%) during pregnancy compared to those born in LAC (Table 1).

**Table 1:** Comparison of characteristics between U.S.-born and LAC-born birthing people in California, 2007–2020

Maternal Characteristics	U.S.-born (*n* = 3,522,173) *n*(%)	LAC-born (*n* = 1,385,679) *n*(%)
Age, mean (SD)	28.5 (6.2)	29.3 (6.2)
BMI (kg/m^2^), mean (SD)	26.7 (6.6)	26.5 (6.2)
Nulliparous	1,500,406 (42.6)	361,911 (26.1)
**Race/Ethnicity**		
Non-Hispanic White	1,332,776 (37.8)	8,393 (0.6)
Hispanic	1,536,993 (43.6)	1,355,775 (97.8)
Non-Hispanic Black	260,873 (7.4)	3,126 (0.2)
Non-Hispanic Asian	168,565 (4.8)	1,765 (0.1)
Other^a^	222,966 (6.3)	16,610 (1.2)
**Education**		
Less than High School	405,063 (11.5)	673,264 (48.6)
High School, or GED equivalent	1,774,944 (50.4)	524,219 (37.8)
College education and above	1,216,722 (34.5)	138,862 (10.0)
Missing	125,444 (3.6)	49,334 (3.6)
**Substance Use Disorder**		
Nicotine or tobacco use during Pregnancy	131,719 (3.7)	4,202 (0.3)
Alcohol use disorder during Pregnancy	14,387 (0.4)	976 (0.1)
Substance use disorder during Pregnancy	93,684 (2.7)	5,216 (0.4)
**Prenatal Care**		
Adequate Plus	980,929 (27.9)	381,330 (27.5)
Adequate	1,552,293 (44.1)	603,939 (43.6)
Intermediate	498,773 (14.2)	182,446 (13.2)
Inadequate	358,572 (10.2)	164,414 (11.9)
Missing	131,606 (3.7)	53,550 (3.9)
**Source of Payment**		
Private insurance	1,936,733 (55.0)	267,921 (19.3)
Public insurance	1,481,183 (42.1)	1,067,153 (77.0)
Other payment^b^	28,967 (0.8)	4,097 (0.3)
Missing	75,290 (2.1)	46,508 (3.4)
**Pre-Existing Conditions**		
Diabetes	37,109 (1.1)	15,094 (1.1)
Hypertension^c^	55,994 (1.6)	12,090 (0.9)

**Footnotes**:

^a^American Indian/Alaska Native, Hawaiian/Pacific Islander, other race group, two or more races, or unknown

^b^Self pay, all other types of pay, no pay, Tricare, or unknown pay

^c^Hypertension without preeclampsia or proteinuria

Variations were observed in education, health insurance, and prenatal care between U.S.-born and LAC-born birthing people. Among LAC-born, 48.6% had less than a high school degree, compared to 11.5% of U.S.-born birthing people. For health insurance, 55.0% of U.S.-born people had had private insurance compared to only 19.3% of LAC-born people. Most LAC-born people (77.0%) had public health insurance, as shown in Table 1. Prenatal care patterns showed similar trends between both groups.

#### LAC-born compared by region of origin

Among the 1,385,679 births to LAC-born people, the majority were from Mexico (82.1%) and Central America (14.3%), with smaller proportions from the Andean-Guiana Shield region (1.8%), Brazil (0.7%), Southern Cone (0.5%), and the Caribbean (0.6%). Most birthing people from Mexico, Central America, and Southern Cone identified as Hispanic, whereas those from Brazil identified as either Hispanic (44.1%) or Non-Hispanic White (42.5%), and the Caribbean identified as either Hispanic (62.8%) or non-Hispanic Black (24.8%) (Table 2). The average age at birth was between 29.1 and 29.3 years for birthing people from Mexico and Central American, and between 31.2 and 33.4 years for all other regions. BMI trends were similar, with Mexico (26.7 kg/m2) and Central America (26.0 kg/m2) in the overweight range. Education attainment varied significantly with Mexico (50.4%) and Central America (48.6%) having a higher proportion of birthing people with less than a high school degree compared to other regions (Andean-Guiana: 7.3%, Brazil: 2.0%, Southern Cone: 4.6%, and Caribbean: 7.0%) (Table 2).

**Table 2:** Comparison of characteristics of LAC-born birthing people by region of origin in California, 2007–2020

	Mexico (*n* = 1,138,029)		Central^1^ (*n* = 198,372)			Andean-Guiana^2^ (*n* = 24,632)			Brazil (*n* = 9,169)	South^3^ (*n* = 6,714)			Caribbean^4^ (*n* = 8,763)	
*Maternal Characteristics*	n(%)		n(%)			n(%)			n(%)	n(%)			n(%)	
Age, mean (SD)	29.3 (6.9)		29.1 (6.0)			32.2 (5.6)			33.4 (4.9)	33.3 (5.3)			31.2 (6.0)	
BMI (kg/m^2^), mean (SD)	26.7 (6.2)		26.0 (6.3)			24.7 (4.9)			23.4 (4.2)	24.2 (5.1)			25.5 (5.8)	
Nulliparous	284,103 (25.0)		55,918 (28.2)			10,506 (42.7)			4,867 (53.1)	2,929 (43.6)				3,588 (40.9)
**Race/Ethnicity**														
Non-Hispanic White	2,076 (0.2)		501 (0.3)			766 (3.1)			3,898 (42.5)	663 (9.9)			489 (5.6)	
Hispanic	1,124,857 (98.8)		193,322 (97.5)			22,613 (91.8)			4,043 (44.1)	5,527 (82.3)			5,413 (61.8)	
Non-Hispanic Black	< 11 (< 0.1)		842 (0.4)			144 (0.6)			25 (0.3)	< 11 (< 0.1)			2,116 (24.2)	
Non-Hispanic Asian	99 (< 0.1)		226 (0.1)			405 (1.6)			558 (6.1)	281 (4.2)			196 (2.3)	
Other^a^	10,989 (1.0)		3,481 (1.8)			704 (2.9)			645 (7.0)	242 (3.6)			549 (6.3)	
**Education**														
Less than High School	573,967 (50.4)		96,389 (48.6)			1,808 (7.3)			184 (2.0)	307 (4.6)				609 (7.0)
High School, or GED equivalent	430,934 (37.9)		74,428 (37.5)			10,329 (41.9)			2,624 (28.6)	2,289 (34.1)				3,615 (41.3)
College education and above	94,362 (8.3)		19,340 (9.6)			11,488 (46.6)			5,705 (62.2)	3,804 (56.7)				4,163 (47.5)
Missing	38,766 (3.4)		8,215 (4.1)			1,007 (4.1)			656 (7.2)	314 (4.7)				376 (4.3)
**Substance Use Disorder**														
Nicotine or tobacco use during Pregnancy	3,384 (0.3)		492 (0.3)			93 (0.4)			69 (0.8)	55 (0.8)				109 (1.2)
Alcohol use disorder during Pregnancy	754 (0.1)		170 (0.1)			25 (0.1)			< 11 (0.1)	< 11 (< 0.1)				14 (0.2)
Substance use disorder during Pregnancy	4,303 (0.9)		674 (0.3)			73 (0.3)			49 (0.5)	23 (0.3)				94 (1.1)
**Prenatal Care**														
Adequate Plus	312,827 (27.5)		53,474 (27.0)			7,307 (29.7)			2,780 (30.3)	2,155 (32.1)				2,787 (31.8)
Adequate	490,419 (43.1)		90,843 (45.8)			11,267 (45.7)			4,397 (48.0)	3,164 (47.1)				3,849 (43.9)
Intermediate	149,562 (13.1)		26,291 (13.3)			3,421 (13.9)			1,256 (13.7)	806 (12.0)				1,110 (12.7)
Inadequate	140,486 (12.3)		20,557 (10.4)			1,872 (7.6)			472 (5.2)	350 (5.2)				677 (7.7)
Missing	44,735 (3.9)		7,207 (3.6)			765 (3.1)			264 (2.9)	239 (3.6)				340 (3.9)
**Source of Payment**														
Private insurance	200,240 (17.6)		38,179 (19.3)			13,628 (55.3)			6,447 (70.3)	4,476 (66.7)				4,951 (56.5)
Public insurance	892,880 (78.5)		156,483 (78.9)			10,041 (40.8)			2,392 (26.1)	2,009 (29.9)				3,348 (38.2)
Other payment^b^	2,941 (0.3)		679 (0.3)			171 (0.7)			69 (0.8)	40 (0.6)				197 (2.3)
Missing	41,968 (3.7)		3,031 (1.5)			792 (3.2)			261 (2.9)	189 (2.8)				267 (3.1)
**Pre-Existing Conditions**														
Diabetes	12,762 (1.1)		2,024 (1.0)			124 (0.5)			53 (0.6)	44 (0.7)				87 (1.0)
Hypertension^c^	9,768 (0.9)		1,887 (1.0)			155 (0.6)			71 (0.8)	60 (0.9)				149 (1.7)

**Footnotes**:

^a^American Indian/Alaska Native, Hawaiian/Pacific Islander, other race group, two or more races, or unknown

^b^Self pay, all other types of pay, no pay, Tricare, or unknown pay

^C^Hypertension without preeclampsia or proteinuria

^1^Costa Rica, El Salvador, Guatemala, Honduras, Nicaragua, and Panama

^2^Venezuela, Bolivia, Colombia, Ecuador, Peru, and Guyana

^3^Argentina, Uruguay, Paraguay, and Chile

There were no significant differences across different substance use disorders and pre-existing conditions. For prenatal care, most individuals received at least intermediate care or higher; however, those born in the Andean-Guiana Shield region and the Caribbean had higher proportions of inadequate care (8%) compared to other regions. Adequate care ranged from 43 to 48% across groups. Health insurance coverage varied, with birthing people from Mexico (78.5%) and Central America (78.9%) primarily having public insurance, whereas other regions had lower proportions of public insurance (Andean-Guiana: 40.8%, Brazil: 26.1%, Southern Cone: 29.9%, and Caribbean: 38.2%) (Table 2).

### Regression results

Overall, the U.S. had a slightly higher prevalence of preeclampsia (4.2%) than LAC. However, the prevalence of GDM was lower in the U.S. (8.0%) compared to LAC (12.8%), with the highest rates among birthing people from Mexico (13.3%), Central America (11.13%), and the Caribbean (10.3%). Preterm births were slightly more prevalent in the U.S. (7.2%) than LAC (7.0%), however, Central America (8.0%) and the Caribbean (8.5%) showed higher proportions. Lastly, for SGA infants, the prevalence was higher among infants born to U.S.-born (9.2%) people compared to infants born to LAC-born people (8.7%), with Central America (10.4%) and Caribbean (10.5%) showing higher proportions.

In the adjusted analysis (Table 3), LAC-born people had lower odds of preeclampsia than did U.S.-born people (AOR: 0.85, 95% Cl: 0.84–0.86). In contrast, LAC-born people had 1.52 times greater odds of having GDM (95% Cl: 1.51–1.53) than U.S.-born people; these odds were slightly elevated among those from Mexico and attenuated yet still significantly elevated among those born in Central America, whereas odds of GDM were lower or null among those born in the Andean-Guiana Shield Region, Brazil, Southern Cone, and Caribbean.

**Table 3:** Association between maternal region of origin and pregnancy outcomes, California 2007–2020

Maternal Place of Birth	Preeclampsia			Gestational Diabetes (GDM)		
	*N* (%)	OR^a^	OR^b^	*N* (%)	OR^a^	OR^b^
		(95%CI)	(95%CI)		(95%CI)	(95%CI)
**Full Sample Population (n = 4908615)**						
U.S.-born (*n* = 3,522,173)	149,381 (4.24%)	*Reference*	*Reference*	259,739 (7.96%)	*Reference*	*Reference*
LAC-born (*n* = 1,385,679)	48,361 (3.49%)	0.82 (0.81–0.83)	0.85 (0.84–0.86)	157,115 (12.79%)	1.61 (1.60–1.62)	1.52 (1.51–1.53)
**U.S.-born vs. LAC-born**,** by region**						
U.S.-born (*n* = 3,522,173)	149,381 (4.43%)	*Reference*	*Reference*	259,739 (7.96%)	*Reference*	*Reference*
Mexico (*n* = 1,138,029)	38,491 (3.50%)	0.79 (0.78–0.80)	0.83 (0.82–0.84)	133,407 (13.28%)	1.67 (1.66–1.68)	1.60 (1.59–1.61)
Central America (*n* = 198,372)	8,382 (4.41%)	1.00 (0.97–1.02)	1.02 (0.99–1.04)	19,870 (11.13%)	1.40 (1.38–1.42)	1.30 (1.28–1.32)
Andean-Guiana (*n* = 24,632)	665 (2.77%)	0.63 (0.58–0.68)	0.63 (0.58–0.68)	1,838 (8.06%)	1.01 (0.97–1.06)	0.76 (0.72–0.79)
Brazil (*n* = 9,169)	276 (3.10%)	0.70 (0.62–0.79)	0.67 (0.60–0.76)	662 (7.78%)	0.98 (0.90–1.06)	0.69 (0.63–0.74)
Southern Cone (*n* = 6,714)	194 (2.98%)	0.67 (0.58–0.76)	0.67 (0.58–0.77)	518 (8.36%)	1.05 (0.96–1.15)	0.75 (0.68–0.82)
Caribbean (*n* = 8,763)	353 (4.20%)	0.95 (0.85–1.05)	0.94 (0.58–0.68)	820 (10.32%)	1.30 (1.21–1.39)	1.02 (0.95–1.10)

^a^Unadjusted odds ratio

^b^Adjusted odds ratio for maternal age and infant year of birth

In unadjusted and adjusted analyses of preterm birth and SGA (Table 4), LAC-born birthing people had infants with lower odds of preterm birth and SGA than did U.S.-born people. When examining by LAC-region, people born in the Caribbean had 1.12 times greater odds of preterm birth (95% CI: 1.04–1.21) and 1.24 times greater odds of SGA birth (95% CI: 1.15–1.33) than did U.S.-born people. Similarly, those born in Central America had 1.16 times greater odds of having SGA (95% CI: 1.15–1.18) than did U.S.-born people.

**Table 4:** Association between maternal region of origin and birth outcomes, California 2007–2020

Maternal Place of Birth	Preterm Births			Small for Gestational Age (SGA)		
	*N* (%)	OR^a^	OR^b^	*N* (%)	OR^a^	OR^b^
		(95%CI)	(95%CI)		(95%CI)	(95%CI)
**Full Sample Population (n = 4908615)**						
U.S.-born (*n* = 3,522,173)	237,430 (7.23%)	*Reference*	*Reference*	297,076 (9.21%)	*Reference*	*Reference*
LAC-born (*n* = 1,385,679)	90,495 (6.99%)	0.97 (0.96–0.97)	0.95 (0.943–0.96)	111,205 (8.73%)	0.95 (0.94–0.95)	0.97 (0.97–0.98)
**U.S.-born vs. LAC-born by region**						
U.S.-born (*n* = 3,522,173)	237,430 (7.23%)	*Reference*	*Reference*	297,076 (9.21%)	*Reference*	*Reference*
Mexico (*n* = 1,138,029)	72,845 (6.84%)	0.95 (0.94–0.95)	0.93 (0.92–0.94)	88,883 (8.47%)	0.92 (0.91–0.93)	0.94 (0.93–0.95)
Central America (*n* = 198,372)	14,695 (8.00%)	1.12 (1.09–1.13)	1.09 (1.07–1.11)	18,649 (10.37%)	1.13 (1.11–1.14)	1.16 (1.15–1.18)
Andean-Guiana (*n* = 24,632)	1,437 (6.20%)	0.86 (0.81–0.90)	0.81 (0.77–0.86)	1,657 (7.21%)	0.78 (0.75–0.82)	0.87 (0.83–0.91)
Brazil (*n* = 9,169)	489 (5.63%)	0.78 (0.71–0.85)	0.73 (0.67–0.80)	716 (8.47%)	0.92 (0.85–0.99)	1.05 (0.97–1.13)
Southern Cone (*n* = 6,714)	345 (5.41%)	0.75 (0.67–0.84)	0.70 (0.63–0.79)	468 (7.49%)	0.81 (0.74–0.89)	0.92 (0.84–1.10)
Caribbean (*n* = 8,763)	684 (8.47%)	1.17 (1.08–1.27)	1.12 (1.04–1.21)	832 (10.49%)	1.14 (1.06–1.22)	1.24 (1.15–1.33)

^a^Unadjusted odds ratio

^b^Adjusted odds ratio for maternal age and infant year of birth

### Mediation analysis

In the mediation analysis, among people born in Mexico, we found that education mediated 23.5% of the association between being born in Mexico and GDM, whereas there was no evidence that prenatal care adequacy or health insurance were contributing to the association (Table 5). For those born in Central America, one-third of the association between being born in Central America and SGA was mediated by payer for delivery, whereas prenatal care and health insurance did not appear to play mediating roles (Table 5). Lastly, among those born in the Caribbean, health insurance mediated approximately 19% of the association between being born in the Caribbean and preterm birth, and among those with infants born with SGA, health insurance accounted for nearly 10% of this association. Prenatal care and education did not mediate the relationship between being born in the Caribbean and preterm birth or SGA (Table 5).

**Table 5:** Analysis of mediating influence on GDM, preterm births, and SGA outcomes among people of LAC

Region	Mediator	Outcome	Direct Effect^1^	Indirect Effect^2^	Proportion Mediated, %^3^
**Mexico**	Prenatal Care	GDM	1.59 (1.58–1.61)	0.99 (0.99-1.0)	1.6
	^#^Education	GDM	1.38 (1.37–1.39)	1.09 (1.08–1.09)	23.5
	Health Insurance	GDM	1.55 (1.53–1.56)	1.02 (1.01–1.02)	4.8
**Central America**	^#^Prenatal Care	GDM	1.30 (1.28–1.32)	0.99 (0.99–0.99)	0.00
	^#^Education	GDM	1.17 (1.15–1.19)	1.03 (1.02–1.05)	18.80
	^#^Health Insurance	GDM	1.33 (1.31–1.36)	0.94 (0.93–0.96)	29.9
	Prenatal Care	SGA	1.16 (1.14–1.18)	1.00 (1.00–1.00)	0.23
	^#^Education	SGA	1.17 (1.14–1.19)	0.99 (0.98–1.01)	3.17
	^#^Health Insurance	SGA	1.11 (1.14–1.22)	1.05 (1.03–1.07)	33.70
**Caribbean**	Prenatal Care	Preterm Birth	1.15 (1.06–1.25)	1.00 (1.00–1.00)	1.60
	^#^Education	Preterm Birth	1.11 (1.02–1.21)	0.99 (0.99-1.00)	0.00
	Health Insurance	Preterm Birth	1.13 (1.04–1.22)	1.03 (1.02–1.03)	19.40
	Prenatal Care	SGA	1.24 (1.15–1.34)	1.00 (0.99-1.00)	0.00
	^#^Education	SGA	1.23 (1.14–1.33)	1.00 (0.99-1.00)	0.16
	Health Insurance	SGA	1.23 (1.15–1.32)	1.02 (1.02–1.02)	9.80

**Footnotes**:

Models adjusted for maternal age and infant year of birth

^1^Effects of region on adverse pregnancy outcomes that are not mediated by each social determinant of health

^2^Effects of region on adverse pregnancy outcomes mediated by each social determinant of health

^3^Proportion of effect of region on adverse pregnancy outcomes mediated by each social determinant of health

^#^Modeled with interaction term between exposure and mediator

## Discussion

In this cohort of nearly 6 million births in California from 2007 to 2020, we documented differences in the odds of adverse pregnancy and birth outcomes by maternal region of origin. Although GDM was elevated for those born in LAC compared to U.S.-born, those findings only extended to birthing people born in Mexico and Central America, while those in Andean-Guiana Shield regions, Brazil and the Southern Cone had lower odds of GDM relative to U.S. born people. For LAC regions with increased odds of GDM, education appeared to partially mediate the relationship. For preterm birth, LAC-born birthing people had slightly lower odds compared to U.S.-born, although, when disaggregated by region, there were higher odds of preterm birth among Caribbean and Central American born birthing people. Similar findings were observed for SGA. Education and health insurance appeared to add to this observed increase in the odds.

Our results are consistent with previous studies indicating greater odds of GDM among those from Mexico compared to the U.S [51, 52]. and with research showing significantly greater odds of preterm birth among infants born to people from Central America and the Caribbean compared to those born in the U.S [1]. However, our findings regarding preeclampsia and SGA diverge from other studies. Whereas we found lower odds of preeclampsia among those from LAC, prior studies have revealed a higher prevalence of preeclampsia among Hispanics born outside the U.S. (9.0%) compared to those born in the U.S. (8.2%) [53], although research in this area remains extremely limited. Additionally, contrary to our findings documenting higher odds for SGA among birthing people from the Caribbean and Central America, previous studies have found lower prevalence among those from LAC overall [1]. However, given the heterogeneity by region, direct comparisons are challenging as different sample proportions from various regions can yield different findings.

Prior studies have shown mixed results compared to ours, likely due to differences in data disaggregation (e.g., ethnicity). Ethnicity-based studies often exclude people from LAC who do not identify as Hispanic (e.g., Brazil, Caribbean), which in turn can reduce the diversity of the study population [54]. Additionally, our study is based on California-births only, whereas the majority of other studies are nationwide or from other states [1–3, 12, 13, 51–53, 55, 56]. Comparing a California-only study with nationwide studies may lead to different findings due to the unique composition of California’s population. For instance, California is a border-state with a diverse immigrant population in which half originates from LAC [6]. Other states such as Texas, Florida, and New York also have numerous LAC-born immigrants; however, their LAC populations are not as large or as predominantly Mexican as California’s, which contributes to the observed differences [5, 57]. A study in Utah observed better birth outcomes among LAC-born birthing people compared to those U.S.-born [13]. In contrast, a study in Texas found a higher risk of GDM among LAC-born birthing people compared to U.S.-born. These studies, however, may have collapsed some or all LAC sub-populations into a single group, potentially overlooking important differences among the various sub-populations. Other nationwide studies have reported mixed results, which could also be due to the collapsing of heterogenous LAC sub-populations.

Research shows that immigrant receiving contexts (e.g., integration, health insurance) as well as pre-migration conditions in places of origin can play important roles in migrant health outcomes. Further, data on pre-migration conditions influencing mobility in administrative cohorts is limited. Migrating birthing people’s decisions can be linked to their migration drivers, destinations, and associated vulnerabilities. For instance, safety and socioeconomic insecurities in Central America, alongside scarce economic opportunities in Mexico, are key drivers of migration to the U.S [58, 59]. Additionally, regions experiencing major crises, such as environmental impacts in the Caribbean, exhibit higher emigration rates [60–64]. These factors, along with early life exposures in native countries, contribute to variations in risk of adverse birth outcomes depending on the composition of the LAC-born sample [17–19, 21, 23–26, 65].

Our study is unique in its approach to disaggregating data based on geographical proximity, which allowed us to categorize LAC into six distinct regions, as opposed to grouping entire continents or ethnicities. While our analysis did not permit examination at the country level due to sample size limitations, our approach still revealed significant heterogeneity in pregnancy and birth outcomes across the identified regions, which is novel finding that suggests the importance of such disaggregation in future research and intervention development.

Our mediation analysis revealed health insurance and education as mediators for most pathways between region of origin and adverse outcomes among people from LAC, underscoring the importance of interventions that address these social determinants of health to advance health equity for LAC-born birthing people in the U.S [46]. Although most U.S.-born and LAC-born birthing people received adequate prenatal care and had health insurance coverage, when analyzed by region, we observed higher proportions of public health insurance among birthing people from Mexico and Central America compared to those from the U.S. and other regions within LAC. In terms of education, U.S.-born birthing people had higher educational attainment than LAC-born. By region, Mexico and Central America, which constitute most of the LAC population, had higher proportions of people with a high school degree or less. Whereas those from the Andean-Guiana Shield, Brazil, Southern Cone, and the Caribbean had lower proportions of high school degree or less and higher proportions of college education and above.

As we reflect on our study, it is important to consider both of its strengths and limitations. A key strength of this study is our use of a population-based administrative dataset including nearly 6 million births in California. This resulted in large, diverse sample, allowing for a counterfactual mediation analysis procedure that maintains its accuracy despite unmeasured confounding between the mediator and outcome, which is a critical step towards understanding the underlying pathways between region of origin and outcomes. Unfortunately, as is common in many population-based studies using administrative data, these do not provide specifics on migration or its social context, including drivers, timing, duration, acculturation, or immigration status [4, 36, 66]. Additionally, our sample of LAC-born is restricted to people who were born outside the U.S. and delivered in California, limiting our analysis to this specific population who came to the U.S., potentially introducing bias related to the ‘healthy migrant’ selection effect [8]. Further, our candidate mediators were selected to reflect socioeconomic and health access disparities that may differ by nativity but ultimately were based on availability in administrative claims. Future studies could include much more holistic capture of mediators that span the life course. Additionally, there is potential for underreporting certain prepregnancy conditions or risks, particularly among people lacking health insurance or facing barriers accessing prenatal care, which may have resulted in misclassification. Finally, we did not account for clustering by region in statistical analysis, potentially underestimating variance in the models.

Future studies examining the relationship between nativity and pregnancy and birth outcomes that collects and analyzes such variables is recommended due to their recognized importance in shaping migrant health access and outcomes [12, 67] and should strive for disaggregated approaches wherever possible. Additionally, future studies should consider data on conditions that pregnant and birthing people experience in countries of origin, during the migration process, or post-arrival that could influence pregnancy and birth outcomes in the U.S., including those specific to Mexico, Central America, and the Caribbean, where many migrants and asylum-seekers experience high exposure to violence, crime, and related socioeconomic factors (e.g., potential food and housing insecurity, intimate partner violence, lack of obstetric care) that may shape perinatal risks [58, 59]. While countries and regions of nativity proxy for many exposures, we were unable to further disaggregate and identify drivers of disparities given the data source. Including data on key early life exposures as mediators such as nutrition, food availability, environmental exposures, and chronic stress would provide a better understanding of their impact on adverse outcomes.

## Conclusion

In this administrative birth cohort of nearly 6 million births, we found significant heterogeneity in adverse pregnancy and birth outcomes among Latin American and Caribbean (LAC)-born individuals by region of origin compared to U.S.-born people. Those from Mexico, Central America, and the Caribbean had an increased risk of adverse pregnancy and birth outcomes, education and health insurance should be further evaluated as intervention targets. Our findings underscore the importance of research into tailored interventions, as different groups have unique challenges and risks for adverse pregnancy outcomes.

## Electronic supplementary material

Below is the link to the electronic supplementary material.

Supplementary Material 1

## Data Availability

The data that support the findings of this study are available from the California Department of Public Health (CDPH). Restrictions apply to the availability of these data, which were used under license for this study. Authors do not have permission to share data. We direct researchers to the CDPH Center for Health Statistics and Information, and the California Department of Health Care Access and Information for information on requesting and accessing California state data.

## Abbreviations

AOR: Adjusted Odds Ratios
APNCU: Adequacy of Prenatal Care Utilization
BMI: Body Mass Index
GDM: Gestational Diabetes Mellitus
HCAI: Health Care Access and Information
ICD: International Classification of Diseases
LAC: Latin America and the Caribbean
SGA: Small for Gestational Age
SOMI: Study of Outcomes in Mothers and Infants
UCSD: University of California, San Diego
U.S.: United States
