# Supplementary material for: Variations in adverse pregnancy and birth outcomes among Latin American and Caribbean-Born birthing people by region of origin, California birth cohort, 2007–2020
Source: BMC Pregnancy Childbirth. 2025 Apr 2;25:384. doi: 10.1186/s12884-025-07483-6 (PMC11963516; doi:10.1186/s12884-025-07483-6)
Supplement: Supplementary file 1 — Supplementary Material 1 [file 12884_2025_7483_MOESM1_ESM.docx]

**Supplemental Table of Contents**

Supplemental Table 1. Data sources of Study of Outcomes in Mothers and Infants

**Supplemental Table 1.** Data sources of Study of Outcomes in Mothers and Infants

| **Variable** | **Data source** | **Notes/ICD codes** |
| --- | --- | --- |
| Race/ethnicity | Birth records | Non-Hispanic White, Black, American Indian/Native American, Asian, Asian-Chinese, Asian-Japanese, Asian-Korean, Asian-Vietnamese, Asian-Cambodian, Asian-Thai, Asian-Laotian, Asian-Hmong, Other, Indian, Filipino, Hawaiian, Guamanian, Samoan, Eskimo, Aleut, Pacific Islander, Withheld/Unknown |
| Maternal age | Birth records | Date of delivery- date of birth (in years) |
| Maternal education | Birth records | Did not attend, 1^st^-11^th^ grade, 12^th^ grade no diploma, HS graduate, GED, Some college, Associate, Bachelor, Masters, Doctorate, Professional |
| Body mass index | Birth records | Calculated from height and pre-pregnancy weight |
| Expected payer for delivery | Birth records | Medi-Cal, Tri-Care, Other government programs, Private insurance, Self-pay, Other, Unknown/Unreported |
| Nulliparity | Birth records | Number of previous live births |
| Nicotine | HCAI, birth records | ICD9 305.1, 649.0. ICD10: O99.33, F17; coded if present in either |
| Substance use disorder diagnosis | HCAI | ICD-9: 648.3, 304, 305.2, 305.3, 305.4, 305.5, 305.5, 305.6, 305.7, 305.8, 305.9 (during pregnancy or at birth admission)  ICD-10 P04.4 (infant), F11, F12, F13, F14, F15, F16, F18, F19 |
| Alcohol use disorder diagnosis | HCAI | ICD9: 303, 305.0. ICD10: F10 |
| Adequacy of prenatal care | Birth records | Kotelchuck Index |
| Preexisting diabetes | HCAI, birth records | ICD9: 648.0, 250. ICD10: E10, E11, E12, E13, E14, O24.0, O24.1, O24.2, O24.3, O24.9; coded if present in either |
| Preexisting hypertension | HCAI, birth records | ICD9: 648.0, 250. ICD10: E10, E11, E12, E13, E14, O24.0, O24.1, O24.2, O24.3, O24.9; coded if present in either |
| Small for gestational age | Birth records | Calculated using birthweight, gestational age, and sex |
| Preterm births | Birth records | Best obstetric estimate |

*HCAI: Health Care Access and Information
